# In Silico Survey and Characterization of *Babesia microti* Functional and Non-Functional Proteases

**DOI:** 10.3390/pathogens10111457

**Published:** 2021-11-10

**Authors:** Monica Florin-Christensen, Sarah N. Wieser, Carlos E. Suarez, Leonhard Schnittger

**Affiliations:** 1 Instituto de Patobiologia Veterinaria (IPVET), Centro de Investigaciones en Ciencias Veterinarias y Agronomicas, Instituto Nacional de Tecnología Agropecuaria (INTA), Hurlingham C1033AAE, Argentina; wieser.sarah@inta.gob.ar (S.N.W.); schnittger.leonhard@inta.gob.ar (L.S.); 2 Consejo Nacional de Investigaciones Científicas y Técnicas (CONICET), Buenos Aires C1033AAJ, Argentina; 3 Animal Disease Research Unit, USDA-ARS, Pullman, WA 99163, USA; suarez@wsu.edu; 4 Department of Veterinary Microbiology and Pathology, Washington State University, Pullman, WA 99163, USA

**Keywords:** human babesiosis, *Babesia microti*, therapeutic drugs, peptidases

## Abstract

Human babesiosis caused by the intraerythrocytic apicomplexan *Babesia microti* is an expanding tick-borne zoonotic disease that may cause severe symptoms and death in elderly or immunocompromised individuals. In light of an increasing resistance of *B. microti* to drugs, there is a lack of therapeutic alternatives. Species-specific proteases are essential for parasite survival and possible chemotherapeutic targets. However, the repertoire of proteases in *B. microti* remains poorly investigated. Herein, we employed several combined bioinformatics tools and strategies to organize and identify genes encoding for the full repertoire of proteases in the *B. microti* genome. We identified 64 active proteases and 25 nonactive protease homologs. These proteases can be classified into cysteine (*n* = 28), serine (*n* = 21), threonine (*n* = 14), asparagine (*n* = 7), and metallopeptidases (*n* = 19), which, in turn, are assigned to a total of 38 peptidase families. Comparative studies between the repertoire of *B. bovis* and *B. microti* proteases revealed differences among sensu stricto and sensu lato *Babesia* parasites that reflect their distinct evolutionary history. Overall, this data may help direct future research towards our understanding of the biology and pathogenicity of *Babesia* parasites and to explore proteases as targets for developing novel therapeutic interventions.

## 1. Introduction

Human babesiosis caused by *Babesia microti* is a malaria-like tick-borne zoonotic disease, first described in the 1950s in the USA, with an increasing number of cases reported ever since in this and other countries around the world [1]. Infections proceed asymptomatic or are accompanied by mild or moderate signs in immunocompetent patients but often lead to severe disease and even death in neonates and the elderly or immunocompromised adults [2].

Some wild rodents act as natural reservoirs of *B. microti*, where the parasite is transmitted both by bites of *Ixodes* sp. ticks, as well as transplacentally [3,4]. Humans are dead-end hosts and suffer accidental infections mainly through tick bites. Transplacental and blood transfusion-related transmissions have also been documented [1,5,6]. 

Currently, there is no specific therapy for *B. microti* human babesiosis [7]. The recommended therapeutic drugs to treat *B. microti* infections are azithromycin plus atovaquone as the first choice or a combination of clindamycin and quinine as an alternative [7,8]. However, the reported appearance of *B. microti* parasites resistant to the first two drugs in chronically infected patients and the negative side effects of the latter two call for the development of alternative therapeutic strategies and increased investments in this field [2,7,8,9,10]. 

*B. microti* belongs to the Apicomplexa phylum and, as such, has a mandatory parasitic lifestyle that alternates between its definitive tick host and its intermediate mammalian hosts. Complex physiological processes and molecular interactions between the pathogen and host are needed for invasion, egress, parasite development in the tick stages, and migration processes that lead to the completion of the parasite life cycle and its efficient perpetuation and dissemination. Among the molecules involved in these events, parasite proteases, i.e., enzymes that catalyze proteolytic cleavages, are bound to be of paramount importance [2,11,12].

Indeed, proteases of the model Apicomplexan protozoans *Toxoplasma gondii* and *Plasmodium falciparum* have been shown to participate in several essential physiological processes, including nutrient acquisition and processing, invasion and egress from host cells, protein recycling, posttranslational processing, and signal transduction, among others [13,14,15]. Due to their vital roles and the fact that they show low or no identity with host-encoded peptidases, parasite proteases have been proposed as potential drug targets and/or vaccine candidates [16,17,18,19,20]. In the case of *Babesia* spp., the importance of peptidases for parasite survival and their potential as therapeutic targets are highlighted by several studies showing that different protease inhibitors significantly impede parasite growth in vitro and/or in vivo [21,22,23,24,25].

The present study aims to shortlist the proteases encoded in the *B. microti* genome by organizing the information available in the MEROPS protease database, as well as identifying additional peptidases by homology searches for paralogs within the *B. microti* genome and orthologs of previously described active proteases of *B. bovis* [26]. We also tested the hypothesis that the repertoires of functional proteases encoded in the genomes of *B. bovis* and *B. microti* differ, possibly due to the peculiarities displayed in their life cycles and their different phylogenetic placements [27,28]. The information recorded in this study can be applied to future research aimed at understanding the biology of this emergent pathogen and designing new therapeutic interventions.

## 2. Results and Discussion

### 2.1. Survey of B. microti Proteases

The present study shows that the *B. microti* genome encodes for at least 64 active proteases and 25 non active protease homologs. These proteases belong to the cysteine (*n* = 28), serine (*n* = 21), threonine (*n* = 14), aspartic (*n* = 7), and metallopeptidase (*n* = 19) types, which, in turn, are assigned to a total of 38 peptidase families (Table 1).

The classification into peptidase types refers to the nature of the nucleophile in the hydrolytic reaction, which can be the thiol of a cysteine in cysteine peptidases, the hydroxyl of a serine, or a threonine residue in serine and threonine peptidases, respectively, or water bound to aspartic acid or to a metal ion in aspartic and metallopeptidases, respectively. An additional protease group has been described, the glutamic peptidases, in which the nucleophile is water bound to a glutamic acid residue, but these enzymes are absent in Apicomplexan protozoa. Peptidases of each type are assigned into families according to sequence similarities. Non active protease homologs are characterized by bearing a conserved protease domain but lacking in the active site one or more of the critical amino acids needed for catalysis [29]. 

Twenty of the proteases presented in this study are not included in the MEROPS database and were identified by homology searches. In addition, five of the proteases listed in Table 1 presented duplicated MEROPS entries, likely because of the use of different sources of peptide sequences in this database (Table 1). On the other hand, a number of *B. microti* proteases have been annotated in GenBank as hypothetical proteins, uncharacterized proteins, or following the designation of another conserved domain also present in the sequences, and they are, thus, not identifiable by searches using keywords such as protease or peptidase. Importantly, despite the exhaustive search carried out to produce the list presented in Table 1, the presence of additional protease-coding genes in the *B. microti* genome that passed inadvertently in this study cannot be ruled out. In addition, it should be noted that the predicted catalytic activity for some threonine proteases (Table 1) could not be determined beyond doubt and needs to be confirmed experimentally.

All of the listed active and non active proteases are transcribed in *B. microti* merozoites, suggesting they likely fulfill a relevant functional role in this parasite stage [30]. In addition, 17 active and non active proteases were identified in the proteomic profile of *B. microti* during the acute infection of a hamster model (Table 1) [31]. Proteases that remained undetected might be expressed in low amounts at the intraerythrocytic stage or bear physicochemical characteristics that preclude detection by the experimental approach employed in this study [31].

Localization predictor algorithms located most of the identified proteases in the cytoplasm. Four proteases were predicted as extracellular and six as lysosomal. The latter might reach the extracellular milieu by the fusion of vacuoles with the plasma membrane, as has been shown for *Tetrahymena thermophila*, a free-living protozoon belonging together with Apicomplexa to Alveolata. However, this mechanism has not been demonstrated for *Babesia* spp. [32]. Other predicted locations include the nucleus, the mitochondria, a plastid (which would correspond to the apicoplast, in this case), the plasma membrane, the endoplasmic reticulum, the Golgi apparatus, and the peroxisomes (Supplementary Table S3). Importantly, these predictions are only tentative until they have been experimentally confirmed. Additionally, the used algorithm is not able to predict the location of proteases in Apicomplexa-specific secretory organelles, such as rhoptries and micronemes, where the trafficking of proteases has been shown to occur in *Plasmodium* and *Toxoplasma* [33].

### 2.2. Aspartic Proteases

Seven aspartic proteases were found in the *B. microti*-predicted proteome, all of which bear the aspartate and, in the case of the A1 family, also the phenylalanine or tyrosine residues in their active sites, needed to display catalytic activity (Table 1). 

Interestingly, a recent transcriptomics study involving four of the five A1 aspartic protease genes of *B. microti* showed stage-associated expression for two of them. Thus, while BmR1_01G02485 (encoding cathepsin E-B or ASP2) displayed higher expression in mouse blood intraerythrocytic stages than in the stages present in *I. ricinus* gut or salivary glands, the opposite was true for BmR1_03g03850 (encoding ASP6). These results suggest a role for ASP2 in processes connected to the asexual reproduction of the parasite and/or gametocyte formation and, for ASP6, in zygote and/or kinete development, kinete dissemination in tick tissues, including salivary glands, and sporogony. For the other two studied A1 aspartic protease-encoding genes, BmR1_04g07350 and BmR1_04g05270, corresponding to ASP3 and ASP5, respectively, expression was similar in the three stages, suggesting a role in invasion both of erythrocytes and tick cells or in other cellular processes such as secretion or the trafficking of proteins [34].

Aspartic proteases have been proposed as chemotherapeutic targets against *B. microti*. Indeed, the aspartic protease inhibitors Lopinavir and Atazanavir, which are well-tolerated drugs used in HIV patients, were shown to be potent suppressors of *B. microti* infection in vitro, as well as in vivo, in a mouse model [24]. It is unknown which parasite aspartic protease is affected by these inhibitors, but one candidate is the signal peptide peptidase (SPP, XP_021338622), which has a critical role in the maintenance of the homeostasis of the endoplasmic reticulum. Consistent with this view, in the case of *P. falciparum*, these inhibitors were effective in blocking SPP activity and in vitro parasite growth [24,35]. Notably, *B. microti* and *Plasmodium* sp. SPP proteins are orthologous (results not shown) but do not have a counterpart in *B. bovis* (Supplementary Table S4) or any other *Babesia* sp. (not shown). 

Hemoglobin is certainly the main protein source available for the nutrition of intraerythrocytic parasites. The sequential steps for hemoglobin degradation by *Plasmodium* sp., as described by Guzman et al. 1994 [36], start with the unwinding of the molecule and partial digestion by aspartic proteases, followed by cysteine protease cleavage, which yields protein fragments that are finally degraded by exopeptidases, generating free amino acids useful for parasite nutrition. In *Plasmodium* sp., the first part of this process takes place in the food vacuole and involves the aspartic proteases Plasmepsins I-IV and is then followed by the action of papain-like cysteine proteases in the erythrocyte cytoplasm [37]. There is no evidence that a food vacuole is present in *B. microti*, and consistent with its absence, Plasmepsins I-IV homologs cannot be found in this parasite. The lack of these enzymes has been used as an argument to postulate that *B. microti* is not able to degrade hemoglobin [38]. However, it may be hypothesized that other *B. microti* aspartyl proteases of the A1 family (Table 1), likely secreted to the erythrocyte cytoplasm, are able to initiate hemoglobin degradation, such as pepsin A (XP_021337801), predicted to have a signal peptide and, thus, be exported to the erythrocyte cytoplasm (Supplementary Table S3). To find out whether this is the case or there is an alternative protein source available for the nutrition of the intraerythrocytic trophozoite and merozoite stages would need experimental exploration.

### 2.3. Cysteine Proteases

Cysteine proteinases are involved in the essential biological roles of Apicomplexan parasites [13,39,40]. They are present in *B. microti* with at least 27 members, of which 18 are predicted to be catalytically active (Table 1).

In *P. falciparum*, the papain-like falcipain-2 and falcipain-3 peptidases of the C1A family have attracted the most attention among cysteine proteases as potential therapeutic targets against malaria [41]. As mentioned above, these enzymes participate in hemoglobin degradation in the intraerythrocytic stage of the parasite, and, in addition, falcipain-2 has been shown to cleave erythrocyte cytoskeletal proteins during egress from the host cell [42,43]. Falcipain-2 orthologs have been characterized in *B. bovis*, *B. bigemina*, and *B. ovis* and have been named bovipain-2, babesipain, and ovipain-2, respectively. Similar to their *P. falciparum* counterpart, they are expressed inside merozoites and also released to the erythrocyte cytoplasm, consistent with the dual role described for falcipain-2 [44,45,46,47]. The significant impairment of the in vitro growth of *B. ovis* and *B. bovis* merozoites by antibodies against ovipain-2 and a papain-like C1A cysteine protease, respectively, indicate a relevant role of this type of enzymes in the propagation of the asexual stages of *Babesia* spp. [47,48].

The *B. microti* ortholog of falcipain-2 (XP_012650559) has four paralogs (three active proteases and one non-protease homolog), one of which is 100% identical (XP_012650562; Table 1). The corresponding genes for these two identical proteins are located on the same strand of chromosome 3, separated by a ~5 kb intergenic region, where two unrelated genes are found in the opposite strand. Predictor algorithms localized XP_012650559 and XP_012650562, either within lysosomes or other vacuoles or secreted through a non classical pathway (Supplementary Table S3). This predicted localization agrees well with that described for their counterparts in *B. bovis*, *B. bigemina*, and *B. ovis* [44,45,46,47,48]. In a recent study, an enzymatically active recombinant form of *B. microti* XP_012650559 (rBmCYP) was expressed in *E. coli*. The activity of rBmCYP against a fluorescent peptide was significantly inhibited by recombinant forms of the cysteine protease inhibitors cystatins 1 and 2 of *Riphicephalus haemaphysaloides* ticks [49]. Although *R. haemaphysaloides* is not a typical *B. microti*-transmitting tick, it has been suggested as a potential vector for this parasite in China [50]. These results coincide with the inhibition exerted by *R. microplus* cystatins on a *B. bovis* C1A cysteine protease and suggest the involvement of these enzymes in tick host–pathogen interactions [51]

Interestingly, the phylogenetic analysis of C1A cysteine protease paralog profiles of piroplasmids of the *Babesia*, *Theileria* and *Cytauxzoon* genera corroborates the assignment of analyzed species into Clades I–VI according to their 18S rRNA gene sequences [27,52].

### 2.4. Serine Proteases 

At least thirteen functional serine proteases and eight non functional protease homologs belonging to nine families are encoded in the *B. microti* genome (Table 1). 

A prominent group of serine proteases is constituted by the S54 family, which consists of rhomboid proteases (ROMs). ROMs were first described in *Drosophila melanogaster* and later shown to be present in all kingdoms of life, fulfilling various relevant roles, including cell signaling in animals, quorum sensing in bacteria, homeostasis regulation in mitochondria, and the dismantling of adhesion complexes in apicomplexan protozoa. They are characterized by having six to seven transmembrane domains and their active site embedded in the lipid bilayer [53,54]. 

ROMs have been thoroughly studied in the apicomplexans *Toxoplasma gondii* and *Plasmodium* spp. The former encodes ROM1–6, according to the nomenclature defined by Dowse and Soldati, 2005 [55], all of which have, with exception of ROM2, homologs in *P. falciparum*. The latter parasite has four additional ROMs that are not present in *T. gondii*, designated ROM7–10 [56,57]. *T. gondii* and *Plasmodium* sp. ROM4 proteases were shown to cleave parasite adhesins, thus dismantling the adhesive junctions formed between the membranes of the host and parasite, a process needed for parasite internalization into the host cell [53,57]. Due to their critical role in invasion, ROMs are regarded as potential targets for therapeutic interventions against apicomplexans [58]. Indeed, two ROM4 inhibitors were shown to specifically block the *P. falciparum* invasion of human erythrocytes [59]. Additionally, experimental vaccine formulations based on *T. gondii* and *Emeria tenella* ROM4 were able to partially protect mice and chickens, respectively, against challenges [20,60]. 

In a recent study, ROM-coding genes were identified in the genomes of several piroplasmids and shown to belong exclusively to the ROM4, ROM6, ROM7, and ROM8 types. While the latter three were always present in a single copy, two to five ROM4 paralogs could be found depending on the piroplasmid lineage analyzed [61]. *B. microti* has two ROM4 paralogs, one of which (XP_021338238) has been misannotated as “ROM3” in GenBank (Table 1). ROM4 proteinases are found exclusively throughout the phylum Apicomplexa, which is consistent with their predicted role in invasion of the host cell, a critical mechanism for these obligate parasites [56]. ROM6, on the other hand, is the only piroplasmid rhomboid not exclusive to apicomplexans and has been shown to participate in various processes, including mitochondrial homeostasis, apoptosis, and the electron transport chain [62]. Accordingly, a mitochondrial localization was predicted for *B. microti* ROM6 (XP_021338360; Supplementary Table S3). *B. microti* ROM7 (XP_012650510) and ROM8 (XP_021338098) were predicted to localize in the membranes of the endoplasmic reticulum and the Golgi apparatus, respectively (Supplementary Table S3). These two types of ROMs are present in *Plasmodium* sp. and piroplasmids but not in other apicomplexans. Their functions are unknown but could be related to processes shared by all Aconoidasida, such as those that take place during the intraerythrocytic stage [61]. Finally, three members of the “derlin” subfamily were found in *B. microti* (Table 1). Derlins are catalytically inactive members of the Rhomboid Superfamily and were first described in yeast and later found in mammals and other organisms. Their function is still unclear, but it has been suggested that they could be part of a channel through which misfolded proteins are retro-translocated from the endoplasmic reticulum to the cytoplasm prior to their ubiquitination and degradation [63]. 

Notably, for *B. bovis*, one of the ROM-encoding genes (XP_001610128) was found to be significantly higher expressed in the parasite stages present in the hemolymph of *Rhipicephalus microplus* ticks as compared to the stages present in bovine blood, suggesting that the role of this protease is mostly associated with the development of the parasite in the tick [64]. It remains to be analyzed whether a similar scenario takes place for the corresponding orthologs in *B. microti* and other piroplasmids. 

In an early study, the serine protease activity of *B. bovis* merozoite homogenates was found to be higher in two virulent than in two avirulent strains from Australia, and thus, these proteases were postulated as virulence determinants [65]. However, in a later study, all the genes encoding for active proteases (*n* = 66) were shown to be present and transcribed to similar levels in the asexual blood stages of a *B. bovis* virulent parental strain and an attenuated strain, obtained by successive blood passages in splenectomized bovines [26]. These data suggest that the virulent/attenuated phenotype in this parasite is not related to a different peptidase gene content or to changes in the transcriptional levels of any peptidase-coding gene. To establish whether or not parasite serine or other types of proteases are virulence determinants in *Babesia* spp. will need further experimental evidence, but in any case, their relevance for pathogenicity is based on the vital role they probably fulfill in the parasitic lifestyle.

### 2.5. Metalloproteases

Metalloproteases contain a metal ion at their active site, which acts as a catalyst in the hydrolysis of peptide bonds, and are represented by at least 17 active and two non active protease homologs in *B. microti* (Table 1) [29]. 

Among them, methionine aminopeptidases (MAPs), which are present with four members in *B. microti* (M24A family, Table 1), take care of the N-terminal methionine excision from polypeptides, general metabolism of amino acids and proteins, and regulation processes that imply the activation and inactivation of biologically active peptides [66]. Inhibitors of MAPs significantly reduced the in vitro growth of *P. falciparum*, *B. bovis*, *B. bigemina*, *B. caballi*, and *T. equi*, highlighting a relevant role of MAPs in the survival of these parasites [23,67]. Moreover, *B. microti*-infected mice treated with MAP inhibitors reached significantly lower parasitemia levels than untreated mice [23]. Additionally, one of the *B. microti* MAPs (XP_012649271) was tested in mice as a potential vaccine candidate for human babesiosis. Immunization with an *E. coli*-expressed recombinant form of this MAP induced a Th1 immune response characterized by IgG2a antibody titers and IFN-γ production, and provided partial protection against the challenge with *B. microti* [68]. Although the number of boosters and protein amounts needed to achieve this effect would not be practical to apply in humans, these results suggest a potential usefulness of MAPs in future vaccine formulations against *B. microti*. 

### 2.6. Threonine Proteases and the Proteasome

The proteasome is a cylindrically shaped large complex of proteins in charge of degrading intracellular proteins destined for destruction that have been tagged with polyubiquitin chains, thereby controlling many cellular processes, such as cell cycle progression and cell signaling [69,70]. All *B. microti* threonine proteases are proteasome constituents (seven alpha and seven beta 20S proteasome subunits, Table 1). Additionally, a metalloprotease with a proteasome regulatory function is also listed among the *B. microti* proteases (XP_021338577 of the M67 family), while assignment of other proteases to this structure needs experimental confirmation. Due to their vital role in cell physiology, drugs targeting proteasome functions have been proposed as therapeutics against several parasitic diseases [71,72,73]. In the case of *Babesia* sp., the proteasome inhibitors epoxyketones and boronic acid were shown to reduce the chymotrypsin activity of the proteasome in lysates of *B. divergens* in vitro cultures, leading to the accumulation of poli-ubiquinated proteins and, also, impeding parasite growth in vitro [24]. One of the epoxyketones, carfilzomib, was also assayed in *B. microti*-infected mice. Carfilzomib is a covalent and irreversible peptide inhibitor of the β5 subunit of the human proteasome approved for the clinical treatment of multiple myeloma [74]. Blood lysates of *B. microti*-infected mice treated with carfilzomib also showed the accumulation of poli-ubiquinated proteins as compared to untreated mice. Moreover, carfilzomib treatment reduced the peak parasitemia levels without apparent toxic effects in the treated mice. Although the dose required to eliminate the parasite would be toxic when applied in humans, these studies indicate that specifically targeting the *B. microti* proteasome would be a possible chemotherapeutic approach against this parasite [24].

### 2.7. Comparison between B. microti and B. bovis Functional Proteases

The genome of *B. microti* is the smallest among Apicomplexans and encodes 7% less genes compared to that of *B. bovis.* This difference is mainly due to the large *vesa* and *SmORF* multigene families present in *B. bovis*, which are absent in *B. microti* [38]. These two gene families encode for highly variable proteins that are involved in escaping the immune system of the vertebrate host and cytoadhesion [75,76]. It remains unknown whether strategies to escape effectors of the immune system exist in *B. microti*. However, cytoadhesion, especially affecting brain capillaries, has not been described as a major pathogenic mechanism for this parasite [2]. Other unraveled differences include the lack of spherical body proteins in *B. microti*, consistent with a reduced apical complex [38]. Additionally, contrary to *B. bovis*, *B. microti* does not have an oligosaccharyl transferase in charge of transferring a (NAcGlc)_2_ moiety from a lipid-linked oligosaccharide to a nascent protein destined for the secretory pathway in the endoplasmic reticulum. Thus, a significant difference among *B. bovis* and *B. microti* is the lack of ability of the latter to produce N-glycosylated proteins [77]. 

In the present study, we hypothesized that the differences between *B. bovis* and *B. microti* include the repertoire of active proteases encoded in their genomes. By orthology searches, we observed that most *B. bovis* active proteases have an ortholog in *B. microti* (Supplementary Table S4). The lack of orthology was connected in all but two cases to the expansion of a protease-coding ancestor gene into different numbers of paralogs, which most likely took place after the separation of the most recent common ancestor (MRCA) of *B. bovis* and *B. microti*, and thus, they differentiate both species. However, the S8 family of serine proteases is present with a single member, a subtilisin-like protein (XP_001610126), only in *B. bovis* but is absent in *B. microti*. The *B. bovis* subtilisin-like protein gene is syntenic with orthologous genes in *B. divergens*, *B. ovata*, and *Babesia* sp. Xinjang (data not shown). Importantly, characterization of the subtilisin-like protein of *B. divergens* showed that it localizes to dense granules and contains neutralization-sensitive B-cell epitopes, consistent with a relevant role in the invasion or establishment of the parasite in the infected erythrocyte, as observed for subtilisin-1 and subtilisin-2 in *P. falciparum* [78,79,80]. The other case is *B. microti* SPP aspartic protease (XP_021338622), which is absent in *B. bovis* and other *Babesia* spp., as mentioned above. The identification of genes absent in *B. microti* and present in other *Babesia* spp. or vice versa can allow comprehending the minimum protein dotation needed to fulfill a basic *Babesia* sp. life cycle, as well as to identify which proteins are associated with species-specific peculiarities and can also be exploited for differential diagnosis, therapeutic, and vaccine developments. *B. bovis* and *B. microti* share important similarities in their life cycles, namely being tick-transmitted and having an asexual reproduction stage exclusively within the erythrocytes of their vertebrate hosts. However, they differ in tick and vertebrate host species, as well as by the presence or absence of transovarial transmission in the tick. Transovarial transmission is, indeed, a trademark of the “true” babesias or *Babesia* sensu stricto, such as *B. bovis*, while those members of the *Babesia* genus that do not have this trait, such as *B. microti*, are considered *Babesia* sensu lato [27,28,81]. These differences are undoubtedly connected with the evolutionary history of *B. bovis* and *B. microti*, which can be clearly visualized by their phylogenetic placement into two distant clades (Clades VI and I, respectively, according to Schnittger et al., 2012 and 2021 [27,28]. 

### 2.8. Non-Peptidase Homologs

At least 25 non-peptidase homologs are encoded in the *B. microti* genome (Table 1). A conserved protease domain can be predicted in their sequences, but they lack one or more of the catalytically relevant amino acids. Non-peptidase homologs are commonly found among living organisms and believed to have evolved from catalytically active enzymes. They have lost their catalytic capacity but developed new functions, such as competitive inhibition regulating their active counterparts or even completely new non-protease-related activities [82,83]. An extreme case of loss of function is observed with a group of paralogs of *B. microti* that include three metalloproteases of the M41 family and 14 other non-protease members. Different from other non-peptidase homologs, the latter do not have a recognizable protease active site region. According to their conserved domains, their functions include the hydrolysis of nucleoside triphosphates, fusion of vesicles, intracellular transport, and proteasome regulation (Supplementary Table S1). 

### 2.9. Conclusions and Perspectives

Proteases are attractive targets against a large number of infectious agents, since many of them are druggable and participate in essential biological processes of pathogenic virus, bacteria, protozoa, and fungi [84]. Indeed, several protease inhibitors are commercially available, and some are successfully employed in the treatment of HIV and Hepatitis C [85,86]. The use of protease inhibitors against other relevant viruses, such as dengue and SARS-CoV-2, has also been postulated [87,88].

The present study was aimed at organizing the available information of *B. microti* proteases and extending the array of identified peptidases encoded in its genome. This information is expected to set the stage for future research directed to understand the biology and pathogenicity of this parasite and to explore proteases as targets for developing novel therapeutic interventions. Recent advances in *B. microti* gene editing will permit exploring the functional relevance of selected proteases [89,90]. In addition, the application of computer-based inhibitor screening and the use of optimized pipelines to test drug efficacies using in vitro cultures and animal models allows obtaining new therapeutics against human babesiosis in a relatively short period of time [34,91,92].

## 3. Materials and Methods

The proteases of *B. microti*, R1 strain, presented in this study were identified by three different search approaches: (i) extracting and organizing the data available for this parasite in the MEROPS database (www.ebi.ac.uk/merops/, accessed on 1 September 2021) [29], (ii) the identification of homologs of *B. bovis* proteases predicted as active, as reported by Mesplet et al. (2011) [26], and (iii) the search for paralogs of *B. microti* proteases identified in (i) and (ii). Orthology between *B. bovis* and *B. microti* proteases was defined using a BLASTp bidirectional best hit (BBH) approach [93]. Paralogs within the *B. microti* genome were determined by BLASTp (blast.ncbi.nlm.nih.gov/Blast.cgi, accessed on 1 September 2021), considering a threshold E value of 0.05. Peptidase domain names and locations were obtained from the Conserved Domains database of the NCBI.

For those proteases included in the MEROPS database and predicted as active, the relevant amino acids of the catalytic site were identified using the data available at this website. For the proteases not included in the MEROPS database, alignments of *B. bovis* and *B. microti* orthologs were carried out by Clustal omega [94] (https://www.ebi.ac.uk/Tools/msa/clustalo/, accessed on 1 September 2021), and the relevant amino acids described for *B. bovis* in MEROPS were manually identified for the corresponding *B. microti* protease. The non-peptidase homologs included those described as such in the MEROPS database. In addition, the peptidases not present in MEROPS were listed as non-peptidase homologs whenever one or more of the catalytically relevant amino acid residues at the homologous positions were missing upon alignment with the sequence of an active proteinase homolog. 

The presence of transcripts and translated proteins in the blood parasite stages was evaluated in PiroplasmaDB [95] (piroplasmadb.org/piro/app, accessed on 10.09.2021) and in the proteomic database provided in Reference [31], respectively. The subcellular location of each protease was evaluated by the presence of a signal peptide (SignalP 5.0 server, www.cbs.dtu.dk/services/SignalP/, accessed on 10 September 2021) [96] and transmembrane domains [97] (TMHMM server, www.cbs.dtu.dk/services/TMHMM/, accessed on 10 September 2021) and using the localization predictor DeepLoc-1.0 [98] (www.cbs.dtu.dk/services/DeepLoc/, accessed on 10 September 2021) with the settings for eukaryotic sequences

## Figures and Tables

**Table 1:** Proteases belonging to the aspartic, cysteine, threonine, serine, and metallopeptidase types encoded by *B. microti*.

Type	Family	Protein Id	GenBank Annotation	Gene Locus	MEROPS Annotation	Type and Position of Peptidase Domain	Active Side Residues (Active Proteases)	Transcriptomic/ Proteomic Data
Aspartic proteases	A1	XP_021337483	Cathepsin E-B	BMR1_01G02485 BBM_I02485	MER1133958—subfamily A1A unassigned peptidases	PTZ00165 aspartyl protease 82-403	D110, Y158, D307	T
		XP_021337801	Pepsin A	BmR1_04g07350 BBM_III07350	MER0383113 MER1136315 subfamily A1A unassigned peptidases	cd05471 pepsin_like 109-417	D128, F173, D324	T
		XP_021338468	Eukaryotic aspartyl protease	BMR1_03g00915 BBM_III00915	MER1142805 MER0383316 subfamily A1A unassigned peptidases	cd05471 pepsin_like 133-473	D160, F205, D373	T
		XP_021338748	Eukaryotic aspartyl protease	BMR1_03g03850 BBM_III03850	MER0384385 subfamily A1A unassigned peptidases	PTZ00165 aspartyl protease 88-401	D106, Y160, D310	T
		XP_021337625	Plasmepsin V	BmR1_04g05270 BBM_III05270	MER0495838 subfamily A1B unassigned peptidases	cl11403 pepsin_retropepsin _like aspartate proteases 167-502	D198, Y253, D388	T
	A22B	XP_021338622	Signal peptide peptidase	BMR1_03g02475 BBM_III02475	MER0323102 subfamily A22B unassigned peptidases	cl01342 Peptidase_A22B Superfamily 27-225	D115, D156	T
	A28	XP_021337501	DNA damage-inducible protein 1	BMR1_01G02675 BBM_I02675	MER0321004 subfamily A28A unassigned peptidases	cd05479 RP_DDI; retropepsin-like domain of DNA damage inducible protein 221-342	D231	T
Cysteine proteases	C1A	XP_021338611	Cathepsin C	BMR1_03g02385 BBM_III02385	MER0345528 Subfamily C1A unassigned peptidases	PTZ00049 cathepsin C-like protein 273-483	Q274, C280, H44, D466	T
		XP_012647584	Cysteine proteinase	BMR1_01G02595 BBM_I02595	MER0701894 Non-peptidase homolog	PTZ00200 cysteine proteinase 296-475	Inactive	T
		XP_012650559	Papain family cysteine protease	BmR1_04g09925 BBM_III09925	MER0344826 subfamily C1A unassigned peptidases	cd02248 Peptidase_C1A 236-444	Q252, C258, H38, N410	T/H
		XP_012650562	Papain family cysteine protease	BmR1_04g09940 BBM_III09940	-	cd02248 Peptidase_C1A 236-444	Q252, C258, H38, N410	T
		XP_012647628	Papain family cysteine protease	BMR1_01G02825 BBM_I02825	MER0345177 Unassigned peptidase	PTZ00200 cysteine proteinase 324-538	Q342, C348, H48, N503	T
	C2	XP_021337703	Calpain family cysteine protease	BmR1_04g06080 BBM_III06080	MER0348343 subfamily C2A unassigned peptidases	cl00051 CysPc Superfamily 72-354	Q96, C102, H293, N313	T
	C12	XP_021337460	ubiquitin carboxyl-terminal hydrolase L3	BMR1_01G02185 BBM_I02185	MER0342930 MER1171398 family C12 non-peptidase homologs	cl08306 Peptidase_C12 Superfamily 8-224	Inactive	T
	C13	XP_012650207	GPI-anchored transamidase	BmR1_04g08080 BBM_III08080	MER0674277 glycosylphosphatidylinositol:protein transamidase	cl00042 CASc Superfamily 391-639	H532, C574	T
	C14	XP_012648342	Caspase domain	BMR1_02g02900 BBM_II02900	MER0393785 subfamily C14B unassigned peptidases	cl00042 CASc Superfamily 48-168	Inactive	T
	C19	XP_012647713	U4/U6.U5 tri-snRNP-associated protein 2	BMR1_01G03245 BBM_I03245	MER0711213 family C19 non-peptidase homologs	cd02669 Peptidase_C19M 158-365	Inactive	T
		XP_012649658	Ubiquitin carboxyl-terminal hydrolase 25	BmR1_04g05260 BBM_III05260	MER0706972 family C19 unassigned peptidases	cl37989 UCH Superfamily 839-1135	N842, C847, H1084, D1105	T
		XP_021338598	Ubiquitin carboxyl-terminal hydrolase	BMR1_03g02275 BBM_III02275	MER0713031 family C19 unassigned peptidases	cl02553 Peptidase_C19 Superfamily 85-383	N94, C100, H342, D358	T
		XP_012649978	Ubiquitin carboxyl-terminal hydrolase 14	BmR1_04g06875 BBM_III06875	MER0346035 family C19 unassigned peptidases	cl02553 Peptidase_C19 Superfamily 80-479	N112, C117, H399, D443	T
		XP_012647380	Ubiquitin carboxyl-terminal hydrolase 7	BMR1_01G01550 BBM_I01550	MER0714894 family C19 unassigned peptidases	cl02553 Peptidase_C19 Superfamily 379-510	Inactive	T/M
		XP_021337689	Ubiquitin carboxyl-terminal hydrolase	BmR1_04g05926 BBM_III05930	MER0710229 family C19 non-peptidase homologs	cl37989 Ubiquitin carboxyl-terminal hydrolase	Inactive	T
		XP_021338067	Ubiquitin carboxyl-terminal hydrolase 5/13	BMR1_02g00955 BBM_II00955	MER0708474 family C19 unassigned peptidases	cl34941 UBP14 Superfamily 184-673	N315, C321, H751, N746	T
	C26	XP_012647696	CTP synthase	BMR1_01G03155 BBM_I03155	-	cl33465 CTP synthase 4-570	C398, H540	T
		XP_021337469	carbamoyl-phosphate synthase// aspartate carbamoyltransferase	BMR1_01G02285 BBM_I02285	-	cl36884 CPSaseII_lrg Superfamily 459-1571	C334, H407	T
	C44	XP_012650079	glucosamine--fructose-6-phosphate aminotransferase	BmR1_04g07400 BBM_III07400	-	cl36542 PTZ00295 super family	C40	T
	C48	XP_012648199	sentrin-specific protease 1	BMR1_02g02160 BBM_II02160	MER0378492 family C48 unassigned peptidases	cl23802 Peptidase_C48 Superfamily 191-358	H279, D298, Q347, C353	T
		XP_021337449	sentrin-specific protease 2	BMR1_01G02005 BBM_I02005	MER0378539 family C48 unassigned peptidases	cl23802 Peptidase_C48 Superfamily 377-660	H453, D583, Q642, C648	T
	C54	XP_021337321	autophagy-related protein 4	BMR1_01G00840 BBM_I00840	-	cl04056 Peptidase family C54 34-256	Y32, C69, D218, H220	T
	C56	XP_012649637	4-methyl-5(b-hydroxyethyl)-thiazole monophosphate biosynthesis	BmR1_04g05155 BBM_III05155	MER0385822 family C56 non-peptidase homologs	cl00020 GAT_1 superfamily 45′207	Inactive	T/H
	C78	XP_021337753	Peptidase family C78	BmR1_04g06690 BBM_III06690	MER0393880 family C78 unassigned peptidases	cl06790 Peptidase family C78 486-677	Y498, C510, D634, H636	T
	C85A	XP_021337245	Ubiquitin thioesterase otu2	BMR1_01G00165 BBM_I00165	MER0743969 subfamily C85A unassigned peptidases	cl9932 OTU Superfamily OTU-like cysteine protease 65-186	D65, C68, H185	T
	C86	XP_021338227	Josephin	BMR1_02g02671 BBM_II02675	MER0399903 family C86 unassigned peptidases	cl20229 Josephin Superfamily 1-121	Inactive	T
	C97	XP_021338702	PPPDE putative peptidase domain	BMR1_03g03275 BBM_III03275	MER0746696 family C97 unassigned peptidases	cl05462 Peptidase_C97 154-283	H193, C274	T
	C115	XP_021337547	Protein FAM63A	BMR1_01G03140 BBM_I03140	MER0933699 family C115 homologs, unassigned	cl04510 MINDY_DUB 4-256	Q21, C27, H 211	T
Threonine proteases	T1A	XP_012647140	20S proteasome subunit alpha 1	BMR1_01G00290 BBM_I00290	MER1091683—subfamily T1A unassigned peptidases	cd03754 proteasome_alpha_type_6 29-150	T38	T/M
		XP_012650489	20S proteasome subunit alpha 2	BmR1_04g09560 BBM_III09560	MER1089221—subfamily T1A unassigned peptidases	cd03750 proteasome_alpha_type_2 61-224	T62	T
		XP_021337746	20S proteasome subunit alpha 3	BmR1_04g06615 BBM_III06615	MER1088544—subfamily T1A unassigned peptidases	PTZ00246 proteasome subunits alpha 32-188	T33	T
		XP_021337745	20S proteasome subunit alpha 4	BmR1_04g06610 BBM_III06610	-	cl00467 proteasome_alpha_type_7 3-209	T32	T
		XP_012649604	20S proteasome subunit alpha 5	BmR1_04g04985 BBM_III04985	MER1363164—subfamily T1A Non-peptidase homologs	Cd03753 proteasome_alpha_type_5 35-204	Inactive	T
		XP_012650085	20S proteasome subunit alpha 6	BmR1_04g07427 BBM_III07427	MER1089259—subfamily T1A unassigned peptidases	cd03749 proteasome_alpha_type_1 36-205	T33	T
		XP_021338656	20S proteasome subunit alpha 7	BMR1_03g02775 BBM_III02775	-	cl00467 Ntn_hydrolase Superfamily 6-214	Inactive?	T
		XP_012648315	20S proteasome subunit beta 1	BMR1_02g02760 BBM_II02760	-	cl00467 Ntn_hydrolase Superfamily 29-268	T31	T
		XP_012649453	20S proteasome subunit beta 2	BMR1_03g04210 BBM_III04210	MER0378485—proteasome subunit beta2	Cd03763 Proteasome_beta_type_7 73-254	T73	T
		XP_012649873	20S proteasome subunit beta 3	BmR1_04g06340 BBM_III06340	MER0376976—proteasome subunit beta 3	cd03759 proteasome_beta_type_3 5-191	Inactive?	T
		XP_012647857	20S proteasome subunit beta 4	BMR1_02g00410 BBM_II00410	-	Cd03758 proteasome_beta_type_2 1-152	Inactive?	T/M
		XP_021338777	20S proteasome subunit beta 5	BMR1_03g04170 BBM_III04170	MER0376387—subfamily T1A unassigned peptidases	cd03761 proteasome_beta_type_5 24-226	T28	T
		XP_012650322	20S proteasome subunit beta 6	BmR1_04g08685 BBM_III08685	MER1090686—subfamily T1A unassigned peptidases	cl00467 Ntn_hydrolase Superfamily 13-197	T13	T
		XP_021337419	20S proteasome subunit beta 7	BMR1_01G01780 BBM_I01780	MER1088514—subfamily T1A non-peptidase homologs	cl00467 Ntn_hydrolase Superfamily 11-150	Inactive	T
Metallo proteases	M01	XP_012648031	aminopeptidase N	BMR1_02g01305 BBM_II01305	MER0335312—M1 aminopeptidase	PRK14015 pepN aminopeptidase N 366-533	E423, Y506 metal ligand(s): H422, H426, E445	T/M
	M3A	XP_021338435	Mitochondrial intermediate peptidase	BMR1_03g00560 BBM_III00560	MER0817287—family M3 unassigned peptidases	cl14813 GluZincin Superfamily Gluzin Peptidase family 49-519	E482 metal ligand(s): H481, H485, E510	T/M
	M16B	XP_021338255	Probable zinc protease PqqL	BMR1_02g02935 BBM_II02935	MER0925233—subfamily M16A unassigned peptidases	COG0612 PqqL Predicted Zn-dependent peptidase 14-164	E40, E116 metal ligand(s): H37, H41, E123	T
		XP_021337876	Mitochondrial processing peptidase	BmR1_04g08505 BBM_III08505	MER0392117—subfamily M16B non-peptidase homologs	COG0612 PqqL Predicted Zn-dependent peptidase 51-251	Inactive	T/H
		XP_021338005	Mitochondrial processing peptidase	BMR1_02g00260 BBM_II00260	MER0391438– MER0764852 mitochondrial processing peptidase beta-subunit	COG0612 PqqL Predicted Zn-dependent peptidase 49-253	E90, E160 metal ligand(s): H87, H91, E167	T/H
	M16C	XP_012650528	peptidase M16 inactive domain containing	BmR1_04g09765 BBM_III09765	MER0393094—subfamily M16C unassigned peptidases	PTZ00432 falcilysin 78-550	E89, E164 metal ligand(s): H86, H90, E203	T
		XP_021338727	Uncharacterized protein C05D11.1	BMR1_03g03610 BBM_III03610	MER0393111—subfamily M16C non-peptidase homologs	COG1026 Cym1 Zn-dependent peptidase, M16 family 54-450	Inactive	T
	M17	XP_021338349	leucyl aminopeptidase	BMR1_02g03960 BBM_II03960	MER0340008—family M17 unassigned peptidases	PRK00913 multifunctional aminopeptidase a154-499	K300,R376 metal ligand(s): A288, D293, D312, H372, Q374	T/H
	M18	XP_021338536	aminopeptidase	BMR1_03g01710 BBM_III01710	MER0340957– MER1122391 aspartyl aminopeptidase	cl14876 Zinc_peptidase_like_Superfamily 12-481	D95, E296 metal ligand(s): H93, D255, E297, D355, H449	T/H
	M24A	XP_021337644	methionyl aminopeptidase	BmR1_04g05525 BBM_III05525	-	PTZ00053 methionine aminopetidase 2 9-447	H199 metal ligand(s): D230, H299, E332,E427	T
		XP_021337770	methionyl aminopeptidase	BmR1_04g06870 BBM_III06870	MER0394794—methionyl aminopeptidase 1	cd1086 MetAP1 Methionine Aminopeptidase 70-312	H143 metal ligand(s): D160, D171, H234, E267,H298	T
		XP_021337427	methionyl aminopeptidase	BMR1_01G01855 BBM_I01855	MER0395783—subfamily M24A unassigned peptidases	cd1086 MetAP1 Methionine Aminopeptidase 146-514	H219 metal ligand(s): D243, D254, H423, E455, E486	T
		XP_012649271	methionyl aminopeptidase	BMR1_03g03300 BBM_III03300	MER0394867—methionyl aminopeptidase 1	PLN03158 methionine aminopeptidase 105-356	H179 metal ligand(s): D196, D207, H270, E303, E334	T
	M24B	XP_012650004	Xaa-Pro aminopeptidase	BmR1_04g07005 BBM_III07005	-	cd01066 X-Prolyl Aminopeptidase	H382,H468,H491 metal ligand(s): D401, D412, H472, E509, E523	T
	M41 *	XP_021338270	Peptidase family M41	BMR1_02g03060 BBM_II03060	MER0363780—PF14_0616 g.p.	TIGR01241 FtsH_fam ATP-dependent metalloprotease FtsH 431-647	E482 metal ligand(s): H481, H485, D558	T/H
		XP_021338301	AFG3 family protein	BMR1_02g03370 BBM_II03370	MER0363828—family M41 unassigned peptidases	TIGR01241 FtsH_fam ATP-dependent metalloprotease FtsH 461-682	E515 metal ligand(s): H514, H518, D581	T/H
		XP_012648901	ATPase family associated with various cellular activities	BMR1_03g01455 BBM_III01455	MER0362824—family M41 unassigned peptidases	TIGR01241 FtsH_fam ATP-dependent metalloprotease FtsH 355-575	E406 metal ligand(s): H405, H409, D484	T
	M48A	XP_012650086	STE24 endopeptidase	BmR1_04g07429 BBM_III07429	MER0347520—subfamily M48A unassigned peptidases	cd07343 M48A_Zmpste24p_lilke Peptidase M48 subfamily A 170-441	E304 metal ligand(s): H303, H307, E382	T/H
	M67	XP_021338577	26S proteasome regulatory subunit N11	BMR1_03g02055 BBM_III02055	MER0393303—subfamily M67A unassigned peptidases	cd08069 MPN_RPN11_CSN5 Mov34/MPN/PAD-1 family proteasomal regulatory protein Rpn11 and signalosome complex subunits CSN5 25-314	E52 metal ligand(s): S113, P115, D126	T
Serine proteases	S1B	XP_021338066	Protease Do-like 9	BMR1_02g00945 BBM_II00945	MER0960997—subfamily S1B unassigned peptidases	Pfam13365Trypsin_2 78-230	H93, D124, S202	T
	S09	XP_021337263	hypothetical protein	BMR1_01G00280 BBM_I00280	-	cl34357 Protease II 105-542	S536, D592, H628	T
		XP_012649807	Alpha/beta hydrolase domain-containing protein 17C	BmR1_04g06005 BBM_III06005	-	cl27027 Fermentation-respiration switch protein FrsA, has esterase activity, DUF1100 family 102-312	S179, D260, H292	T
		XP_021338600	alpha/beta hydrolase, putative	BMR1_03g02286 BBM_III2290	-	Fermentation-respiration switch protein FrsA, has esterase activity, DUF1100 family 120-256	S142, D194, H246	T
		XP_012650025	alpha/beta hydrolase domain-containing protein 17B	BmR1_04g07110 BBM_III07110	-	cl27027 Fermentation-respiration switch protein FrsA, has esterase activity, DUF1100 family 35-220	S125, D189, H217	T
	S12	XP_012649063	aarF domain-containing kinase	BMR1_03g02265 BBM_III02265	MER1005027—family S12 non-peptidase homologs	cl21491 Transpeptidase superfamily 554-737	Inactive	T
	S14	XP_012648206	ATP-dependent Clp protease, protease subunit	BMR1_02g02195 BBM_II02195	MER0359175—family S14 unassigned peptidases	Cd07017 S14_ClpP_2 Caseinolytic protease (ClpP) 41-228	S135, H160, D209	T
		XP_021337686	Clp protease	BmR1_04g05887 BBM_III05890	MER0359717 family S14 non-peptidase homologs	cd07017 caseinolytic protease (ClpP) 50-227	Inactive	T
	S16	XP_012649081	Lon protease homolog 1 mitochondrial	BMR1_03g02350 BBM_III02350	MER0361396—family S16 unassigned peptidases	cl36736 Ion endopeptidase La 835-1038	S946, K989	T/H
	S26	XP_021338290	mitochondrial inner membrane protease subunit 1	BMR1_02g03240 BBM_II03240	MER1047726—subfamily S26A non-peptidase homologs	Cd06530 S26_SPase_I 64-102	Inactive	T
		XP_012650493	signal peptidase, endoplasmic reticulum-type	BmR1_04g09580 BBM_III09580	MER0334095—signalase (animal) 21 kDa component	cl10465 Peptidase_S24_S26 Superfamily 52-169	S63, H101	T/M
	S33	XP_012648716	cardiolipin-specific phospholipase	BMR1_03g00525 BBM_III00525	-	cl21494 Abhydrolase_1 98-327	S172, D292, H350	T
	S54	XP_021338360	Rhomboid-like protease 6	BMR1_02g04085 BBM_II04085	MER1084044—family S54 unassigned peptidases	cl21536 Rhomboid Superfamily 363-474	S391, H452	T
		XP_021338239	ROM4	BMR1_02g02777 BBM_II02780	MER0374041—family S54 unassigned peptidases	cl21536 Rhomboid Superfamily 177-339	S270, H322	T
		XP_021338238	ROM3 (a)	BMR1_02g02776 BBM_II02775	-	c21536 Rhomboid Superfamily 205-337	S273, H325	T
		XP_012650510	hypothetical protein BmR1_04g09675 (b)	BmR1_04g09675 BBM_III09675	MER1083102—family S54 non-peptidase homologs	cl21536 Rhomboid Superfamily 139-357	S287, H342	T
		XP_021338098	hypothetical protein BMR1_02g01230 (c)	BMR1_02g01230 BBM_II01230	-	cl21536 Rhomboid Superfamily 267-458	Inactive	T
		XP_012647608	Der1-like family	BMR1_01G02725 BBM_I02725	-	cl21536 Rhomboid Superfamily 11-202	Inactive	T
		XP_012650093	Der1-like family	BmR1_04g07462 BBM_III07462	-	cl21536 Rhomboid Superfamily 87-295	Inactive	T/H
		XP_012649979	Derlin 2/3	BmR1_04g06880 BBM_III06880	-	cl21536 Rhomboid Superfamily 12-205	Inactive	T
	S59 *	XP_012650001	Nucleoporin autopeptidase	BmR1_04g06990 BBM_III06990	MER1071845—family S59 non-peptidase homologs	pfam04096 Nucleoporin2 444-579	Inactive	T

Protease types are color-coded: blue: aspartic, light yellow: cysteine, orange: threonine, gray: metallo, and yellow: serine proteases, with darker colors for peptidases predicted as active. The relevant amino acid positions in the active site needed for catalytic activity are included for each active protease. Inactive proteases have a predicted protease domain but lack one or more of the functional amino acids. Paralog groups within each family are underlined. When more than a paralog group is present in a family, different underlining styles are used for each group. (a) ROM4, (b) ROM7 (active protease, wrongly predicted as inactive in MEROPS), and (c) ROM8 [29]. (*) Paralogs of the M41 and the S59 families lacking peptidase domains are shown separately (Supplementary Tables S1 and S2). T: Transcribed genes in the intraerythrocytic stage [30]. H or M: High or medium levels of protein expression detected in Reference [31].

## Data Availability

Not applicable.

## Supplementary Materials

The following are available online at https://www.mdpi.com/article/10.3390/pathogens10111457/s1, Table S1: M41 metalloprotease paralogue family of *B. microti*, Table S2: S59 paralogue family of *B. microti*, Table S3: Predicted subcellular localization of *B. microti* proteases, Table S4: Comparison between the repertoire of *B. microti* and *B. bovis* proteases predicted as active at least in one of either species.
