# Supplementary material for: In Silico Survey and Characterization of Babesia microti Functional and Non-Functional Proteases
Source: Pathogens. 2021 Nov 10;10(11):1457. doi: 10.3390/pathogens10111457 (PMC8621943; doi:10.3390/pathogens10111457)
Supplement: Supplementary file 1 [file pathogens-10-01457-s001.zip › pathogens-1406294-supplementary.pdf]

A) Suppl. Table S1. M41 metalloprotease paralogue family of *B. microti*. Paralogues of XP_021338270 were ientified by pBLAST searches within the *B. microti* predicted proteome ($E_{value} < 0.05$). Only the first three of the list bear an metalloprotease domain and are predicted to have metalloprotease catalytic activity, while for the remaining such a domain cannot be predicted and likely fulfill other cellular functions, according to the conserved domains they possess.

Protein ID	GenBank annotation	Metalloprotease domain	Other conserved domains	Possible functions
XP_021338270	Peptidase family M41	Yes	No	Metalloprotease
XP_012648901	ATPase family associated with various cellular activities (AAA)	Yes	No	Metalloprotease
XP_021338301	AFG3 family protein	Yes	- pfam06480: FtsH Extracellular	Metalloprotease<br>Control of correct folding of proteins
XP_012648808	26S proteasome regulatory subunit T1	No	-COG1222: RPT1, ATP-dependent 26S proteasome regulatory subunit	Proteasome regulation: Posttranslational modification, protein turnover, chaperones
XP_012647944	26S proteasome regulatory subunit T5	No		

XP_021338610	26S proteasome regulatory subunit T6	No		
XP_021337282	26S proteasome regulatory subunit T4	No		
XP_021337577	26S proteasome regulatory subunit T3	No	PTZ00454; 26S protease regulatory subunit 6B-like protein; Provisional	Proteasome regulation?
XP_021338764	26S proteasome regulatory subunit T2	No	cl33178; 26 proteosome regulatory subunit 4-like protein; Provisional	
XP_021337444	Cell division cycle protein 48 homolog	No	cl36852; AAA family ATPase, CDC48 subfamily	Budding and transfer of membrane from the ER to the Golgi apparatus
XP_021338213	RIX7, NVL, ribosome biogenesis ATPase	No		
XP_021338827	transitional endoplasmic reticulum ATPase	No		
XP_012647478	Cell division cycle protein 48 homolog MJ1156	No		
XP_021337191	vacuolar protein-sorting-associated protein 4	No	- cl36852; AAA family ATPase, CDC48 subfamily	Budding and transfer of membrane from ER to Golgi

			-cl38936: P-loop containing Nucleoside Triphosphate Hydrolases<br><br>- cl00299: MIT (microtubule interacting and transport)	Hydrolysis of nucleoside triphosphates<br><br>Binding to endosomal sorting complexes required for transport of microtubules
XP_012650057	microtubule-severing ATPase	No	-cl38936: P-loop containing Nucleoside Triphosphate Hydrolases<br><br>-pfam17862: AAA+ lid domain<br><br>-cl32576: DNA-binding ATP-dependent protease La	Hydrolysis of nucleoside triphosphates<br><br>Protease?
XP_021337583	vesicle-fusing ATPase	No	-cl38936: P-loop containing Nucleoside Triphosphate Hydrolases<br><br>- cl36852; AAA family ATPase, CDC48 subfamily<br><br>- smart01073: Cell division protein 48 (CDC48) N-terminal domain<br><br>-cl34016: MoxR-like ATPase	Hydrolysis of nucleoside triphosphates<br><br>Fusion of vesicles

XP_012648107	ATPase family AAA domain-containing protein 3	No	- cl37828: DUF3523 super family (unknown function)<br><br>-cl38936: P-loop containing Nucleoside Triphosphate Hydrolases	Hydrolysis of nucleoside triphosphates<br><br>Unknown additional functions

A) Suppl. Table S2. S59 paralogue family of *B. microti*. Paralogues of XP_012650001were identified by pBLAST searches within the *B. microti* predicted proteome ($E_{value} < 0.05$). In two members of this family, a protease domain cannot be predicted. These proteins are predicted to be localized in the nucleus (Suppl. Table 2 and to form part of nuclear pore complexes.

Protein ID	GenBank annotation	Serine protease domain	Other conserved domains	Possible functions
XP_012650001	Nucleoporin autopeptidase	Yes	pfam13634 Nucleoporin FG repeat region	Peptidase
XP_012649223	Nucleoporin FG repeat region	No	cl41472: ser_rich_anae_1 super family	Part of nuclear pore complexes
XP_021338605	Nucleoporin FG repeat region	No	- pfam13634: Nucleoporin_FG<br>-cl32064: PRK04778 super family<br>-cl37900: Nucleoporin_FG2 super family	Part of nuclear pore complexes

A) Supplementary Table S3: Predicted subcellular localization of *B. microti* proteases. The possible localization of each protease was evaluated by the presence of a signal peptide (SignalP), number of transmembrane domains (TMHMM) and the DeepLoc predictor algorithm.

Type	Family	Protein id.	Genbank annotation	Topology		Localization (DeepLoc)	
				SignalP	TMHMM	Compartment	Soluble or Membrane
Aspartic proteases	A1	XP_021337483	Cathepsin E-B	Yes	0	Lysosome/Vacuole	Membrane
		XP_021337801	Pepsin A	Yes	0	Extracellular	Soluble
		XP_021338468	Eukaryotic aspartyl protease	No	0	Extracellular	Soluble
		XP_021338748	Eukaryotic aspartyl protease	No	1	Lysosome / Vacuole	Soluble

		XP_021337625	Plasmepsin V	Yes	0	Plastid	Soluble
	A28	XP_021337501	DNA damage-inducible protein 1	No	0	Cytoplasm	Soluble
	A22B	XP_021338622	Signal peptide peptidase	No	8	Endoplasmic reticulum	Membrane
Cysteine proteases	C1A	XP_021338611	Cathepsin C	Yes	0	Extracellular	Soluble
		XP_012647584	Cysteine proteinase	No	1	Lysosome/Vacuole	Membrane
		XP_012650559	Papain family cysteine protease	No	1	Lysosome/Vacuole	Membrane

		XP_012650562	Papain family cysteine protease	No	1	Lysosome/Vacuole	Membrane
		XP_012647628	Papain family cysteine protease	No	1	Lysosome/Vacuole	Membrane
	C2	XP_021337703	Calpain family cysteine protease	No	0	Cytoplasm	Soluble
	C12	XP_021337460	ubiquitin carboxyl-terminal hydrolase L3	No	0	Cytoplasm	Soluble
	C13	XP_012650207	GPI-anchored transamidase	Yes	1	Endoplasmic reticulum	Membrane
	C14	XP_012648342	Caspase domain	No	0	Cytoplasm	Soluble
	C19	XP_012647713	U4/U6.U5 tri-snRNP-associated protein 2	No	0	Nucleus	Soluble

		XP_012649658	Ubiquitin carboxyl-terminal hydrolase 25	No	0	Cytoplasm	Soluble
		XP_021338598	Ubiquitin carboxyl-terminal hydrolase	No	0	Nucleus	Soluble
		XP_012649978	Ubiquitin carboxyl-terminal hydrolase 14	No	0	Cytoplasm	Soluble
		XP_012647380	Ubiquitin carboxyl-terminal hydrolase 7	No	0	Cytoplasm	Soluble
		XP_021337689	Ubiquitin carboxyl-terminal hydrolase	No	0	Nucleus	Soluble
		XP_021338067	Ubiquitin carboxyl-terminal hydrolase 5/13	No	0	Cytoplasm	Soluble

	C26	XP_012647696	CTP synthase	No	0	Cytoplasm	Soluble
		XP_021337469	carbamoyl-phosphate synthase// aspartate carbamoyltransferase	No	0	Cytoplasm	Soluble
	C44	XP_012650079	glucosamine--fructose-6-phosphate aminotransferase	No	0	Cytoplasm	Soluble
	C48	XP_012648199	sentrin-specific protease 1	No	0	Nucleus	Soluble
		XP_021337449	sentrin-specific protease 2	No	0	Nucleus	Soluble
	C54	XP_021337321	autophagy-related protein 4	No	0	Mitochondrion	Soluble

	C56	XP_012649637	4-methyl-5(b-hydroxyethyl)-thiazole monophosphate biosynthesis	No	0	Plastid	Soluble
	C78	XP_021337753	Peptidase family C78	No	0	Cytoplasm	Soluble
	C85A	XP_021337245	Ubiquitin thioesterase otu2	No	0	Nucleus	Soluble
	C86	XP_021338227	Josephin	No	0	Nucleus	Soluble
	C97	XP_021338702	PPPDE putative peptidase domain	No	0	Plastid	Soluble
	C115	XP_021337547	Protein FAM63A	No	0	Cytoplasm	Soluble
Threonine proteases	T1A	XP_012647140	20S proteasome subunit alpha 1	No	0	Cytoplasm	Soluble

		XP_012650489	20S proteasome subunit alpha 2	No	0	Golgi apparatus	Membrane
		XP_021337746	20S proteasome subunit alpha 3	No	0	Nucleus	Soluble
		XP_021337745	20S proteasome subunit alpha 4	No	0	Cytoplasm	Soluble
		XP_012649604	20S proteasome subunit alpha 5	No	0	Cytoplasm	Soluble
		XP_012650085	20S proteasome subunit alpha 6	No	0	Cytoplasm	Soluble
		XP_021338656	20S proteasome subunit alpha 7	No	0	Cytoplasm	Soluble

		XP_012648315	20S proteasome subunit beta 1	No	0	Cytoplasm	Soluble
		XP_012649453	20S proteasome subunit beta 2	No	0	Mitochondrion	Soluble
		XP_012649873	20S proteasome subunit beta 3	No	0	Cytoplasm	Soluble
		XP_012647857	20S proteasome subunit beta 4	No	0	Cytoplasm	Soluble
		XP_021338777	20S proteasome subunit beta 5	No	0	Cytoplasm	Soluble
		XP_012650322	20S proteasome subunit beta 6	No	0	Cytoplasm	Soluble

		XP_021337419	20S proteasome subunit beta 7	No	0	Peroxisome	Soluble
Metallo proteases	M01	XP_012648031	aminopeptidase N	No	0	Plastid	Soluble
	M3A	XP_021338435	mitochondrial intermediate peptidase	No	0	Mitochondrion	Soluble
	M16B	XP_021338255	Probable zinc protease PqqL	No	0	Mitochondrion	Soluble
		XP_021337876	Mitochondrial processing peptidase	No	0	Mitochondrion	Soluble
		XP_021338005	Mitochondrial processing peptidase	No	0	Mitochondrion	Soluble
	M16C	XP_012650528	peptidase M16 inactive domain containing	No	0	Cytoplasm	Soluble

		XP_021338727	Uncharacterized protein C05D11.1	No	0	Cytoplasm	Soluble
	M17	XP_021338349	leucyl aminopeptidase	No	0	Plastid	Soluble
	M18	XP_021338536	aminopeptidase	No	0	Cytoplasm	Soluble
	M24A	XP_021337644	methionyl aminopeptidase	No	0	Cytoplasm	Soluble
		XP_021337770	methionyl aminopeptidase	No	1	Mitochondrion	Soluble
		XP_021337427	methionyl aminopeptidase	Yes	0	Mitochondrion	Soluble

		XP_012649271	methionyl aminopeptidase	No	0	Cytoplasm	Soluble
	M24B	XP_012650004	Xaa-Pro aminopeptidase	No	0	Cytoplasm	Soluble
	M41*	XP_021338270	Peptidase family M41	No	0	Mitochondrion	Membrane
		XP_021338301	AFG3 family protein	No	0	Mitochondrion	Membrane
		XP_012648901	ATPase family associated with various cellular activities	No	1	Endoplasmic reticulum	Membrane
	M48A	XP_012650086	STE24 endopeptidase	No	7	Endoplasmic reticulum	Membrane

	M67	XP_021338577	26S proteasome regulatory subunit N11	No	0	Cytoplasm	Soluble
Serine proteases	S1B	XP_021338066	Protease Do-like 9	No	0	Mitochondrion	Soluble
	S09	XP_021337263	hypothetical protein	No	0	Mitochondrion	Soluble
		XP_012649807	Alpha/beta hydrolase domain-containing protein 17C	No	0	Endoplasmic reticulum	Membrane
		XP_021338600	alpha/beta hydrolase, putative	No	0	Nucleus	Membrane
		XP_012650025	alpha/beta hydrolase domain-containing protein 17B	No	0	Nucleus	Soluble
	S12	XP_012649063	aarF domain-containing kinase	No	0	Peroxisome	Membrane

	S14	XP_012648206	ATP-dependent Clp protease, protease subunit	No	1	Mitochondrion	Soluble
		XP_021337686	Clp protease	Yes	0	Extracellular	Soluble
	S16	XP_012649081	Lon protease homolog 1 mitochondrial	No	0	Mitochondrion	Soluble
	S26	XP_021338290	mitochondrial inner membrane protease subunit 1	No	0	Golgi apparatus	Membrane
		XP_012650493	signal peptidase, endoplasmic reticulum-type	No	1	Endoplasmic reticulum	Membrane
	S33	XP_012648716	cardiolipin-specific phospholipase	No	0	Cytoplasm	Soluble

		XP_021338360	Rhomboid-like protease 6	No	3	Mitochondrion	Membrane
		XP_021338239	ROM4	No	7	Golgi apparatus	Membrane
		XP_021338238	ROM3	No	6	Golgi apparatus	Membrane
	S54	XP_012650510	hypothetical protein BmR1_04g09675	Yes	3	Endoplasmic reticulum	Membrane
		XP_021338098	hypothetical protein BMR1_02g01230	No	6	Golgi apparatus	Membrane
		XP_012647608	Der1-like family	No	4	Endoplasmic reticulum	Membrane

		XP_012650093	Der1-like family	Yes	2	Mitochondrion	Membrane
		XP_012649979	Derlin 2/3	No	4	Endoplasmic reticulum	Membrane
	S59	XP_012650001	Nucleoporin autopeptidase	No	0	Nucleus	Soluble

A) Suppl. Table S4. Comparison between the repertoire of *B. microti* and *B. bovis* proteases predicted as active at least in one of either species. Proteases predicted as active in one and inactive in the other species are marked with an asterisk and shaded in lighter colors. Orthology was evaluated by the BLASTp bidirectional best hit method.

Proteases	*Babesia bovis*	*Babesia microti*	Orthologous	*B. bovis* only	*B. microti* only
Aspartyl					
A1	XP_001611483	XP_021337483	x		
A1	XP_001610711	XP_021337801	x		
A1	XP_001610888	XP_021338748	x		
A1		XP_021338468			x
A1	XP_001610957			x	
A1	XP_001611299	XP_021337625	x		
A22		XP_021338622			x
A28	XP_001612143	XP_021337501	x		
Cysteine					
C1A	XP_001608716	XP_021338611	x		
C1A	XP_001609546			x	
C1A	XP_001612131			x	
C1A	XP_001610695	XP_012650559	x		
C1A		XP_012647628			x
C1A		XP_012650562			x
C2	XP_001611895	XP_021337703	x		
C12	XP_001612184	XP_021337460*	x		

C13	XP_001608682	XP_012650207	x		
C19	XP_001611300	XP_012649658	x		
C19	XP_001611747	XP_021338598	x		
C19	XP_001611991	XP_012649978	x		
C19	XP_001609338	XP_012647380*	x		
C19	XP_001610364	XP_021338067	x		
C26	XP_001610053	XP_012647696	x		
C26	XP_001611491	XP_021337469	x		
C44	XP_001609193	XP_012650079	x		
C48	XP_001609932	XP_012648199	x		
C48		XP_021337449			x
C54	XP_001610073	XP_021337321	x		
C78	XP_001612028	XP_021337753	x		
C85	XP_001612242	XP_021337245	x		
C97	XP_001610596	XP_021338702	x		
C97	XP_001609705			x	
Threonin					
T1A	XP_001609757	XP_012647140	x		
T1A	XP_001609795	XP_012650322	x		
T1A	XP_001610115	XP_012648315	x		
T1A	XP_001610788	XP_012649453	x		
T1A	XP_001610822	XP_021338777	x		
T1A	XP_001609200	XP_012650085	x		
T1A	XP_001611166	XP_012650489	x		
T1A	XP_001608800	XP_012649604*	x		

T1A	XP_001612044	XP_021337746	x		
Metallo					
M01	XP_001610522	XP_012648031	x		
M03A	XP_001611394	XP_021338435	x		
M16B	XP_001609640	XP_021338255	x		
M16B	XP_001609291	XP_021338005	x		
M16C	XP_001610012	XP_012650528	x		
M17	XP_001609968	XP_021338349	x		
M18	XP_001611075	XP_021338536	x		
M24A	XP_001611362	XP_021337644	x		
M24A	XP_001611993	XP_021337770	x		
M24A	XP_001609360	XP_021337427	x		
M24A	XP_001610600	XP_012649271	x		
M24B	XP_001611965	XP_012650004	x		
M41	XP_001609615	XP_021338270	x		
M41	XP_001611654	XP_021338301	x		
M41	XP_001611107	XP_012648901	x		
M48A	XP_001609199	XP_012650086	x		
M67A	XP_001610266	XP_021338577	x		
Serine					
S01B	XP_001610362	XP_021338066	x		
S08A	XP_001610126			x	
S09A	XP_001609758	XP_021337263	x		
S09X	XP_001611738	XP_021338600	x		
S09X	XP_001611915	XP_012649807	x		

S09X	XP_001610498	XP_012650025	x		
S14	XP_001609930	XP_012648206	x		
S16	XP_001611731	XP_012649081	x		
S26A	XP_001609075	XP_021338290*	x		
S26B	XP_001611162	XP_012650493	x		
S33	XP_001610202	XP_012648716	x		
S54	XP_001609020	XP_021338360	x		
S54	XP_001610111			x	
S54	XP_001610112			x	
S54	XP_001610113			x	
S54		XP_021338238			x
S54	XP_001610125			x	
S54	XP_001610128	XP_021338239	x		
S54	XP_001611188	XP_012650510*	x		
